# Bouveret’s Syndrome

**DOI:** 10.7759/cureus.4414

**Published:** 2019-04-09

**Authors:** Jobin Philipose, Hafiz M Khan, Moiz Ahmed, Pretty S Idiculla, Sherif Andrawes

**Affiliations:** 1 Internal Medicine, Staten Island University Hospital - Northwell Health, Staten Island, USA; 2 Gastroenterology and Hepatology, Staten Island University Hospital - Northwell Health, Staten Island, USA; 3 Gastroenterology, Icahn School of Medicine at Elmhurst Hospital Center, Elmhurst, USA; 4 Gastroenterology, Staten Island University Hospital - Northwell Health, Staten Island, USA

**Keywords:** bouveret syndrome, gallstone, pneumobilia, intestinal obstruction

## Abstract

Bouveret syndrome is a very rare form of gastric outlet obstruction following the passage of a gallstone from the gallbladder to the duodenum or pylorus through a bilioenteric fistula. We present a unique case of a 78-year-old male complaining of right upper quadrant abdominal pain and who was found to have a gallstone in the proximal duodenum along with pneumobilia and cholecysto-duodenal fistula suggestive of Bouveret’s syndrome.

## Introduction

Bouveret’s syndrome is a rare variant of gallstone ileus, which is caused by the impaction of a gallstone in the duodenum. The gallstone erodes through the wall of gallbladder and duodenum creating a bilioenteric fistula due to persistent inflammation in the surrounding area and pressure necrosis. We report a case of Bouveret’s syndrome in an elderly male who presented with a gastric outlet obstruction. 

## Case presentation

**Figure 1:**
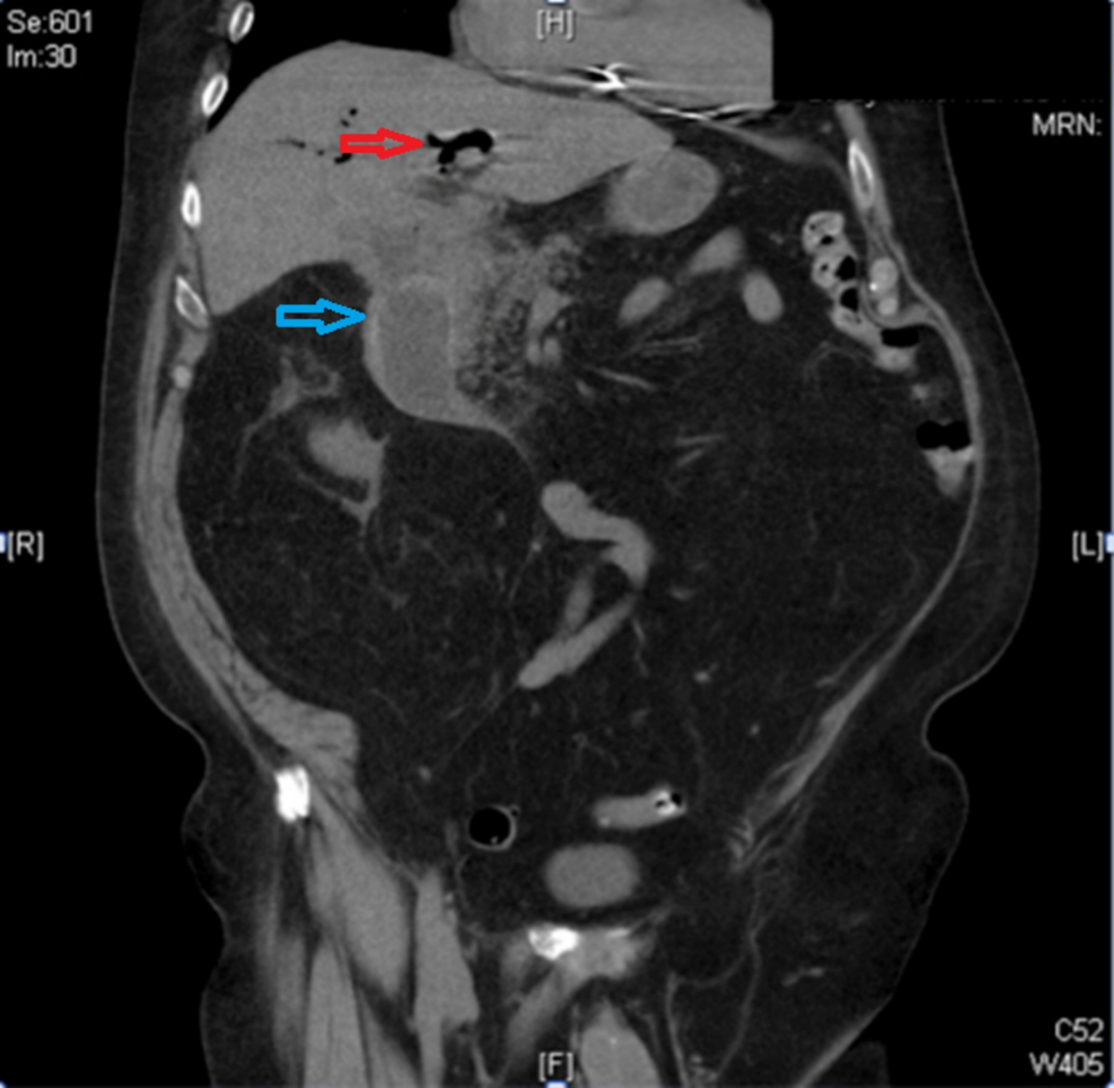
Computed tomography (CT) of the abdomen and pelvis showing impacted gallstone in the duodenum (blue arrow) and pneumobilia (red arrow)

A 78-year-old Caucasian man presented to the emergency room with a sudden onset of severe, intermittent, cramping right upper quadrant abdominal pain with non-bloody, non-bilious vomiting for one day. His personal history included atrial fibrillation and atrioventricular block with an implanted pacemaker. On examination, the abdomen was soft with mild epigastric tenderness, decreased bowel sounds and distension. Labs revealed leukocytosis of 12.46 TH/MM3, alkaline phosphatase of 321 IU/L, aspartate aminotransferase of 52 IU/L, alanine aminotransferase of 47 IU/L, total bilirubin of 1.8 mg/dl, and serum lipase of 161 U/L. An abdominopelvic computed tomography (CT) revealed a 6.6 x 4.4 cm gallstone in the proximal duodenum with surrounding inflammation, cholecysto-duodenal fistula, and pneumobilia (Figure 1).

**Figure 2:**
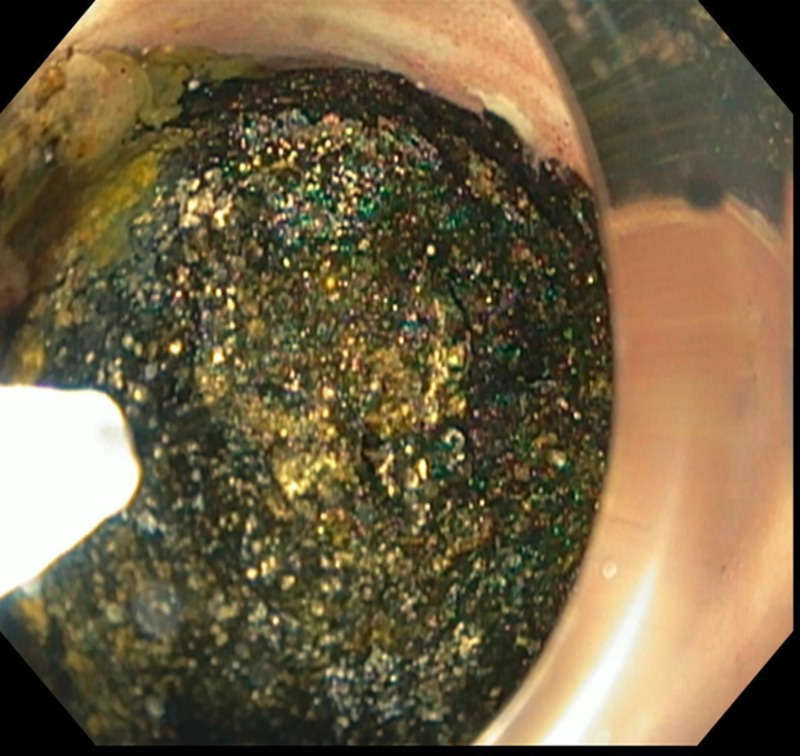
Upper endoscopy showed a large impacted stone in the second portion of the duodenum

Upper endoscopy was performed revealing complete obstruction of the duodenum due to the impacted stone (Figure 2).

**Figure 3:**
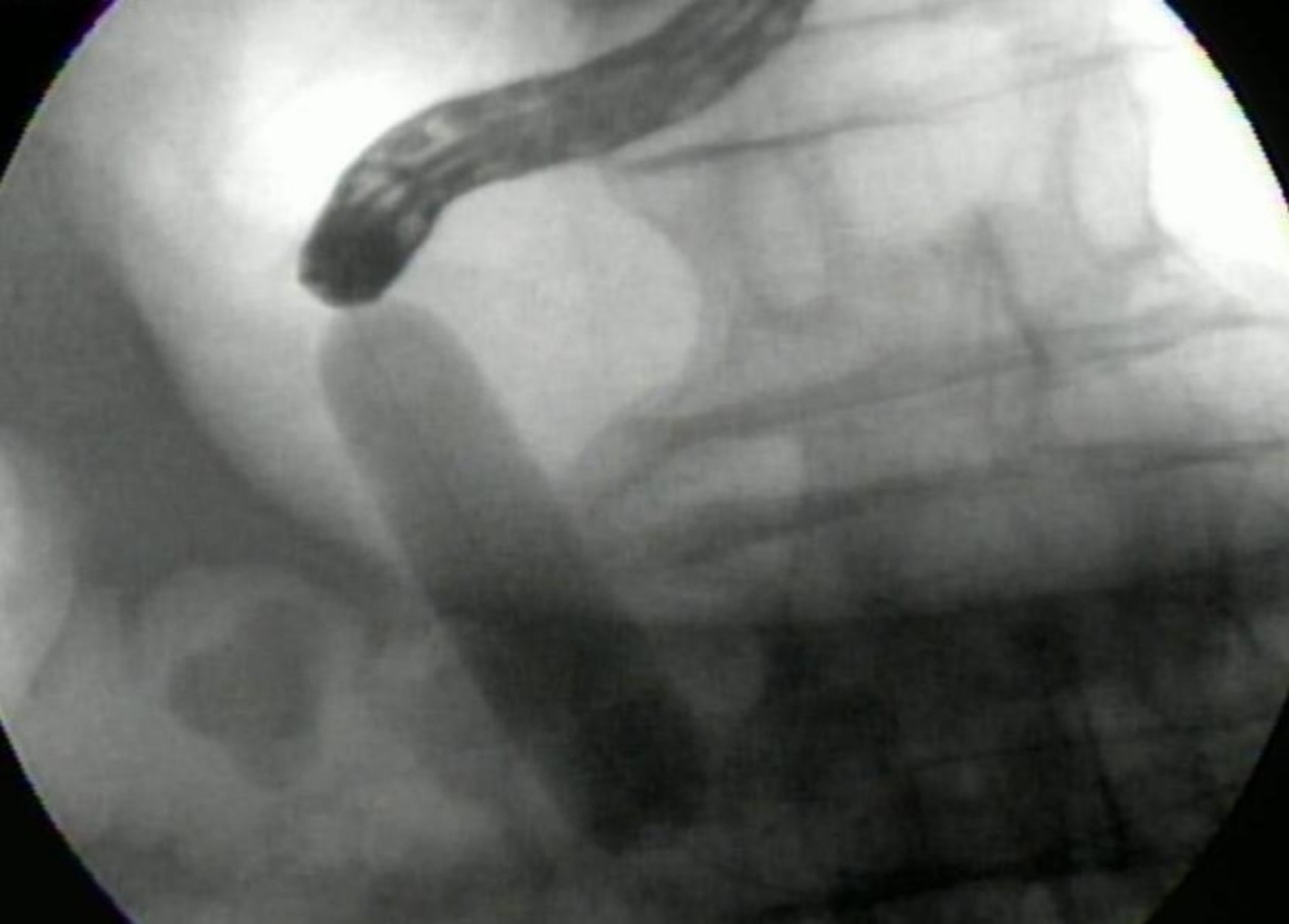
Attempted stone extraction using controlled radial expansion (CRE) balloon

Several endoscopic foreign body and stone retrieval devices, as well as lithotripsy, were attempted to remove or fragment the stone. However, the stone was impacted and exceedingly larger than the available endoscopic retrieval devices. Another attempt was made to inflate the controlled radial expansion (CRE) dilation balloon beyond the impacted stone and drag the stone into the stomach for fragmentation, but it was unsuccessful (Figure 3).

**Figure 4:**
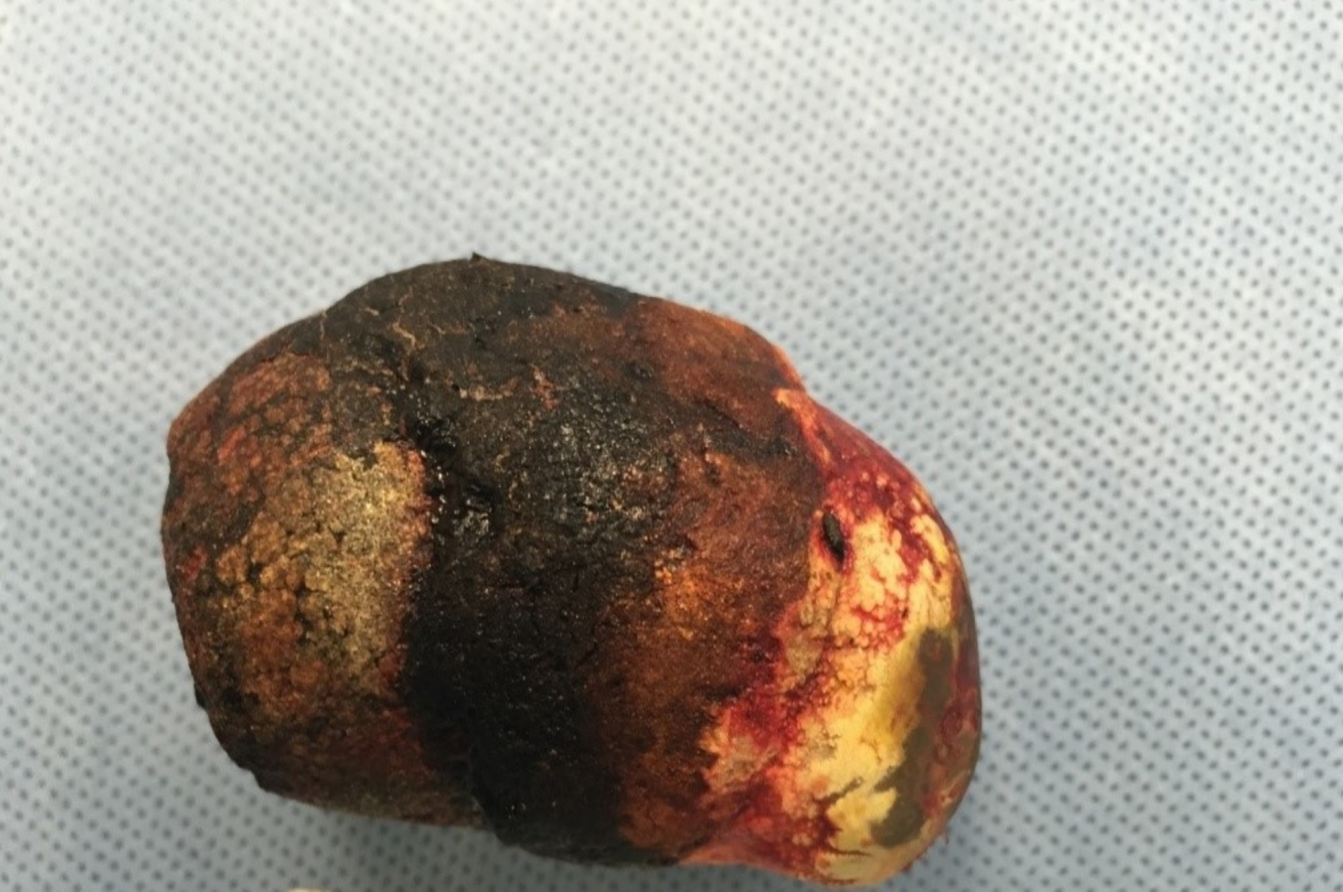
Gallstone removed by duodenotomy

Endoscopic guided electrohydraulic lithotripsy (EHL) was performed, which led to partial fragmentation of the stone. We were able to create a tunnel through the stone but was unable to break the outer shell despite using multiple probes at high power. Laparoscopy was then attempted although he eventually required laparotomy due to adhesions. The stone was successfully extracted through duodenotomy as seen in Figure 4, followed by closure of the cholecysto-duodenal fistula, cholecystectomy, and placement of a temporary feeding gastrojejunostomy tube. The postoperative course remained uneventful, and the patient was discharged after four days.

## Discussion

Gallstone ileus causes intestinal obstruction in 1%-4 % of the cases, of which Bouveret’s syndrome comprises of only 1%-3%. This rare syndrome was first described in 1896 by Leon Bouveret, a French physician, which since then bears his name [1]. It occurs more commonly in elderly women with a median age of 74 years [2]. This is a clinically distinct entity owing to proximal gastrointestinal obstruction, usually caused by large (usually >2.5cm) gallstone [3].

The clinical presentation is nonspecific, varying from gastric outlet obstruction as seen in our case, to acute pancreatitis, upper gastrointestinal bleeding, duodenal perforation, Boerhaave's syndrome and gastric bezoars formation [4-10]. A classic Rigler’s triad of dilated stomach, pneumobilia, and ectopic stone appearing as a filling defect in the duodenum seen on a CT scan is virtually pathognomonic of the Bouveret’s syndrome [11]. This triad is diagnostic in only 21% of the cases with a conventional radiograph [12]. Nevertheless, 15%-25% of the times gallstones are difficult to identify on a CT as they are isodense to adjacent liquid [11]. Thus, high clinical suspicion with appropriate diagnostic imaging helps make the diagnosis easier.

The primary therapeutic goal is gallstone retrieval. Majority of the patients with Bouveret’s being elderly, with a high incidence of concomitant disorders, do carry a high risk of morbidity and mortality post-surgery. Thus esophagogastroduodenoscopy (EGD) plays a significant role both as a diagnostic and therapeutic modality and must be considered as a first line treatment despite its low success rate [13]. There have been case reports of successful retrieval of gallstones of up to 3 cm using endoscopy [14-15]. The different endoscopic methods described are mechanical lithotripsy, laser lithotripsy, extracorporeal shockwave lithotripsy, and intracorporeal electrohydraulic lithotripsy as used in our report [16-17]. The choice of procedure mainly depends upon the availability at the individual center and the endoscopist. A major drawback of all these techniques the risk of dislodgment of the stone fragment distally, causing small bowel obstruction [16].

Despite these advances, surgery is needed in most of the patients with this disease. Surgery can be done laparoscopically or through laparotomy. As in our case, laparoscopic surgery was initially attempted, but it was not successful due to adhesions. Surgical options include enterolithotomy or gastrostomy with or without gallbladder removal and repair of the fistula [13,18]. There is a paucity of data supporting simple surgical stone extraction as oppose to simultaneous cholecystectomy and fistula closure.

## Conclusions

In conclusion, Bouveret’s syndrome is a rare entity and remains a diagnostic and therapeutic challenge. An initial endoscopic trial must always be performed. In cases requiring surgical intervention, a tailored approach must be considered based on individual age and co-morbidities in relation to the morbidity and mortality rates of each approach.
